# Personalized hypertension treatment recommendations by a data-driven model

**DOI:** 10.1186/s12911-023-02137-z

**Published:** 2023-03-01

**Authors:** Yang Hu, Jasmine Huerta, Nicholas Cordella, Rebecca G. Mishuris, Ioannis Ch. Paschalidis

**Affiliations:** 1 Department of Electrical and Computer Engineering, Division of Systems Engineering, Boston University, 8 Saint Mary’s St., Boston, MA 02215 USA; 2 Department of Biomedical Engineering, Faculty of Computing & Data Sciences, Hariri Institute for Computing and Computational Science & Engineering, Boston University, 8 Saint Mary’s St., Boston, MA 02215 USA; 3 Department of Medicine, Boston Medical Center, School of Medicine, Boston University, Boston, MA USA

**Keywords:** Machine learning, Hypertension prescription, Clinical decision support

## Abstract

**Background:**

Hypertension is a prevalent cardiovascular disease with severe longer-term implications. Conventional management based on clinical guidelines does not facilitate personalized treatment that accounts for a richer set of patient characteristics.

**Methods:**

Records from 1/1/2012 to 1/1/2020 at the Boston Medical Center were used, selecting patients with either a hypertension diagnosis or meeting diagnostic criteria (≥ 130 mmHg systolic or ≥ 90 mmHg diastolic, n = 42,752). Models were developed to recommend a class of antihypertensive medications for each patient based on their characteristics. Regression immunized against outliers was combined with a nearest neighbor approach to associate with each patient an affinity group of other patients. This group was then used to make predictions of future Systolic Blood Pressure (SBP) under each prescription type. For each patient, we leveraged these predictions to select the class of medication that minimized their future predicted SBP.

**Results:**

The proposed model, built with a distributionally robust learning procedure, leads to a reduction of 14.28 mmHg in SBP, on average. This reduction is 70.30% larger than the reduction achieved by the standard-of-care and 7.08% better than the corresponding reduction achieved by the 2nd best model which uses ordinary least squares regression. All derived models outperform following the previous prescription or the current ground truth prescription in the record. We randomly sampled and manually reviewed 350 patient records; 87.71% of these model-generated prescription recommendations passed a sanity check by clinicians.

**Conclusion:**

Our data-driven approach for personalized hypertension treatment yielded significant improvement compared to the standard-of-care. The model implied potential benefits of computationally deprescribing and can support situations with clinical equipoise.

**Supplementary Information:**

The online version contains supplementary material available at 10.1186/s12911-023-02137-z.

## Background

Hypertension continues to be a leading factor of death and severe health complications [1]. It can cause a series of cardiovascular disorders, such as ischemic heart disease [2], stroke, and heart failure [3]. While nearly half of the adults with hypertension in the U.S. have Systolic Blood Pressure (SBP) over 140 mmHg, only 24% of these hypertensive patients manage to bring their hypertension under control [4]. The major causes of uncontrolled hypertension are complicated, since inappropriate or inadequate prescriptions, patient noncompliance, and high therapy cost can all lead to treatment failure [5]. Unlike other medical conditions, therapy for hypertension is easily influenced by peripheral components in the biological network [6]. Genetic predisposition, physiological systems, and time-varying environmental factors all play important roles in the pathophysiology of hypertension [7].

Despite the consensus that different patient characteristics and distinct underlying mechanisms of high blood pressure would lead to differential responses towards antihypertensive drugs, personalized hypertension treatment has not been widely adopted in clinical practice. Most clinicians are still following evidence-based clinical guidelines that utilize more generalized standard therapies [6]. There is a need to incorporate various considerations in clinical decision making, such as, medication interactions, comorbidities, white coat effects (i.e., the transient pressor rise triggered by standard BP measurement in the clinical environment) [8, 9], obesity, pseudo-hypertension, and personal lifestyle choices including food and alcohol intake [10, 11]. However, the unknown underlying traits for each patient remain elusive to decipher and account for simply by manually integrating various data sources. Consequently, precise personalized treatment is difficult to attain given the considerable variety of antihypertensive drug options. Existing studies that model hypertension prescriptions are either restricted to analysis in certain circumstances and do not provide full personalization [12] or are limited to certain hypertension prescription type [13].

This study proposes a personalization approach for hypertension treatment based on Machine Learning (ML) algorithms, seeking to maximize the effectiveness of hypertensive medications at the individual level. Being increasingly adopted in the cardiovascular field, ML is expected to facilitate the precision and personalization of hypertension therapies [14]. However, there are several challenges for ML-driven personalization:

1. *The sparse and incomplete nature of the Electronic Health Records (EHRs)* [15–17]. Integrating heterogenous data from different sources, the process of medical data collection is always complex. This usually leads to data ‘outliers’ caused by entry errors, incomplete information, and irregular lab tests, which can significantly reduce the accuracy of ML models. To the best of our knowledge no work on personalized treatment has dealt with ‘outliers’.
2. *Personalized recommendations under sparse patient history*. To make personalized recommendations, an ML model cannot see into the future and can only rely on the *prediction* of counterfactual outcomes under each prescription. Only when the prescription recommended by the model is the same as the administered one, we have ground truth assessment of the outcome. There are several models built for personalized chronic disease treatment using a so-called *contextual bandits* approach [18, 19], which can learn from historical data of the same individual and make predictions. However, this methodology highly relies on frequent patient visits and rich historical information that is hard to find in sparse EHRs. There is a need to make recommendations even for patients with sparse history.
3. *Interpretability of the ML prescriptive model*. While ML is superior to traditional approaches in handling large amounts of data at our disposal, ML adoption in health-care settings is limited by the lack of interpretability and comprehensiveness. Clinicians and patients cannot make medical decisions just based on a black-box model designed and evaluated in a narrower scenario, which may not have access to all information about a specific patient [20]. It is important for the decision makers to understand not just the mechanism of the algorithm, but how and why the model proposes each option. For instance, recent work has developed hypertension treatment prediction models using Deep Learning (DL) methods [21, 22]. While DL models often result in accurate predictions, it is hard to interpret and rationalize the results. Moreover, studies show that for harmonized EHRs data, a DL model did not show superior benefits compared with traditional methods [23].

In this paper, our objective is to design a model able to generate a personalized hypertension prescription based on each individual patient profile, including demographics, vital signs, past medical history, and clinical test records. To that end, we predict the future SBP by using the past outcomes of patients who had the most similar profiles. We adopt a *robustified* regression procedure to immunize predictions against outliers in the EHR data that could bias the result [24]. Our algorithm is based on a regression-informed K-Nearest Neighbor (KNN) [25] approach. Unlike other non-linear approaches such as DL or random forests [26], this method is more reliable than a simple linear model but maintains interpretability and comprehensibility. The predictive power of the prescriptive model has been shown to satisfy certain theoretical guarantees [27]. We incorporated a randomized prescriptive policy to add robustness to the model and only adopt the recommended prescription if the SBP improvement over the previous regimen exceeds a certain threshold.

To help the prescribers interpret the model, we randomly sampled 350 cases, listed the recommended prescriptions, and the corresponding patient profiles. Moreover, we summarized affinity profiles built by the model. These cases were manually reviewed by clinicians at Boston Medical Center (BMC) to make sure that the prescriptive model is capable of maintaining a balance between contraindications and antihypertensive efficacy.

## Materials and methods

### Dataset

We extracted data from Boston Medical Center (BMC) Electronic Health Records (EHRs) from January 1, 2012, to January 1, 2020. The dataset included all patients who satisfied one or more of the following conditions: (i) patients who had a hypertension diagnosis; (ii) patients with high blood pressure in the problem list included in their record; and (iii) patients with recorded SBP measurement exceeding 130 mmHg or a Diastolic Blood Pressure (DBP) measurement exceeding 90 mmHg. We identified 42,752 such patients who also met the following criteria:

- Patients have in the system at least 2 SBP measurements in 180 days (2 defined periods).
- Received at least one type of cardiovascular medication, including Calcium Channel Blockers (CCBs), Thiazide diuretics, Angiotensin Receptor Blockers (ARBs), ACE inhibitors (ACEi), Beta-blockers, Loop diuretics, and Mineralocorticoid-Receptor-Antagonists (MRAs).

### Features

For each qualified patient, we collected their demographics (age, sex, race, language, marital status, ZIP code), past blood pressure records, past medical history, vital signs, symptoms, medication history, laboratory tests results, cigarette usage, information for their social needs and depression test scores. Information on social needs was extracted from the ‘THRIVE’ survey program implemented at BMC. Specifically, THRIVE is a custom screening program created by BMC which surveys patients on their unmet social needs in eight different domains: transportation, ability to secure caregiving for family members, ability to pay for utilities, education, food, housing, employment issues, and ability to pay for medications. All records were de-identified before analysis. The study was approved by the Boston University Medical Campus and Boston University Institutional Review Boards.

### Records timeline design

We split patients’ treatment history based on ‘visits’ to reflect the effect of prescriptions. Each ‘visit’ is defined to occur every 90 days from the patient’s first SBP record in our observation period (2012–2019) until the last recorded SBP measurement. A ‘visit’ is therefore a periodic review period of the patient’s health status rather than an actual visit to the clinic. The SBP measurements, vital signs and all other time-varying features were all captured and averaged over each visit (90-day period). The current prescription of each visit is the drug (or drugs, if multiple) used during the current period. The future outcome is defined as the averaged SBP measurement in mmHg 90 to 180 days after the current visit (i.e., during the next or the following visit). This time lag is chosen because previous studies have defined drug persistence as consistently refilling antihypertensive prescriptions in the subsequent clinic visits within 90–180 days of a previous dispensing [28–30]. Patient visits without valid future outcomes are dropped. In total, we obtained 432,096 valid visits and defined 144 features for each visit.

### Prescriptive policy design

Our prescriptive model considers monotherapy of 7 types of antihypertensive drugs: Calcium Channel Blockers (CCBs), Thiazide diuretics, Angiotensin II Receptor Blockers (ARBs), ACE Inhibitors (ACEi), Beta-Blockers, Loop diuretics, Mineralocorticoid Receptor Antagonists (MRAs). We did not consider specific combinations of drugs as an option because drug combinations are often associated with complex medical constraints and complications, and there are many different combinations one would need to consider. Effective antihypertensive drug combinations usually require all the classes having different and complementary mechanisms of actions [31]. Instead, the model provides a list of recommended agents and their associated confidence probabilities, leaving to the physician the option of prescribing a combination of agents in the recommended list.

The patient’s current prescription is also included in the menu of options for the future prescription. Since frequently changed prescriptions can cause concerns about high health care costs and slow transient periods, we opted to maintain the current treatment where possible. The new prescription will be considered only if the improvement on SBP control is over a pre-specified threshold. Specifically, as detailed in [24, 32], if the expected improvement of the proposed prescriptions on the current SBP is not substantial, the current prescription is adopted instead of the proposed one. A threshold of SBP reduction above which the improvement is considered substantial enough is determined in [24, 32] using certain distributional assumptions on the predicted SBP.

To achieve best performance on future SBP reduction, the most straightforward idea is to choose the drug that yields the lowest predicted SBP. However, this approach may let potential prediction errors mislead clinicians and discard comparable alternatives that could be a better selection in some cases. As a result, we opted for a randomized recommendation policy that prescribes a drug with a probability inversely proportional to its exponentiated predicted SBP. For example, the prescription model may suggest Drug A with probability ${p}_{A}$, Drug B with probability ${p}_{B}$, and Drug C with probability ${p}_{C}$. These probabilities can be interpreted as confidence levels, or as the likelihood that the recommended drug will lead to the minimal future SBP. This has the effect of improving the robustness of the model by exploration and showing to the physician all agents with a significant likelihood of leading to the minimum future SBP. It has been shown statistically that such randomized strategy can achieve nearly optimal future outcomes if an appropriate parameter is chosen [24, 32].

For the purpose of this analysis, a few common relative contradictions and disease specific preferences were incorporated in our prescriptive model. All the rules followed the professional suggestions from BMC clinicians and are based on ACC/AHA hypertension treatment guidelines [4]:

- If pulse < 60, do not use Beta-Blockers;
- If potassium > 4.5, do not use ACEi or ARBs;
- If creatinine of 2 or greater, do not use ACEi or ARBs or Thiazide diuretics;
- If potassium > 5, do not use MRAs;
- If patient has diabetes, should likely be on an ACEi or ARBs;
- If patient has systolic heart failure, should likely be on a Beta-Blocker.

### Models and evaluation scheme

We developed ML models to predict optimal prescriptions for patients with hypertension. A prediction-based prescriptive model was developed to analyze outcomes under each possible medication. The goal is to find the treatment that minimizes future SBP based on the medical history of a group of similar patients. Our methods incorporate K-Nearest Neighbors (K-NN) on a regression-weighted metric, which can capture the similarity between patients’ most predictive characteristics and model the future SBP accordingly. For example, if we want to estimate a patient’s future SBP under Thiazide diuretics, we first train a regression model with all patient visits that were under Thiazide diuretics to predict the future SBP. Suppose this regression model fits a set of coefficients ${\varvec{\beta}}=({\beta }_{1},..., {\beta }_{p})$ to predictive variables ${\varvec{x}}=({x}_{1},..., {x}_{p})$ and predicts the SBP of a patient as $y=\sum_{i=1}^{p}{x}_{i}{\beta }_{i}$, where $p$ is the number of variables used for the prediction. Then, the coefficients ${\varvec{\beta}}$ of the regression model are used to identify the importance of each feature in predicting the SBP outcome. The absolute values of coefficients form weights in measuring the distance between this patient and all the other patient visits that were under Thiazide diuretics. More specifically, we define a distance metric between two patients characterized by predictive variables $\varvec{x} = \left( {x_{1} , \ldots , x_{p} } \right)$ and $\varvec{z} = \left( {z_{1} , \ldots , z_{p} } \right)$ by

$$\left| {\left| {\varvec{x} - \varvec{z}} \right|} \right|_{\varvec{\beta}} = \sqrt {\mathop \sum \limits_{i = 1}^{p} (x_{i} - z_{i} )^{2} \beta_{i}^{2} } ,$$

and use this metric to find the K nearest neighbors to any particular patient with features ${\varvec{x}}$ under Thiazide diuretics. Once the closest visits from the treatment group are identified, we consider their SBP values at their next visit (say $y_{1} \ldots , y_{K}$) and use their average, given by $\hat{y}\left( \varvec{x} \right) = \frac{1}{K}\sum\nolimits_{i = 1}^{K} {y_{i} }$, to estimate the SBP of the patient with features ${\varvec{x}}$ at her next visit. The same procedure is repeated for all the prescription options included in the model. Let M the total number of prescription options and denote by $\hat{y}_{m} \left( {\varvec{x}} \right)$ the predicted next-visit SBP for a patient with features ${\varvec{x}}$ under prescription m. Then, prescription m is recommended for this patient with probability $e^{{ - \xi \hat{y}_{m} \left( \varvec{x} \right) }} /\sum\nolimits_{i = 1}^{M} {e^{{ - \xi \hat{y}_{j} \left( \varvec{x} \right)}} }$, where ξ is a tunable parameter.

Four (regression) algorithms were compared to test the best performance in future SBP reduction: Distributionally Robust Linear Regression (DRLR)-informed KNN (the proposed model), Ordinary Least Square Regression (OLS)-informed KNN, the Least Absolute Shrinkage and Regression Operator (LASSO) regression [33], and Classification and Regression Trees (CART) [34]. The new regularized regression technique DRLR was developed based on Distributionally Robust Optimization (DRO) with an ambiguity set built using a Wasserstein metric [24]. In short, regression is formulated as a game, where an adversary picks a probability distribution from the ambiguity set and expected loss is evaluated using that distribution. The modeler then selects model parameters to minimize that worst-case expected model loss. The approach can mitigate the impact of outliers by hedging against a family of probability distributions that are close to the empirical distribution of the training data. Various theoretical results in [24] establish such robustness properties and establish probabilistic performance guarantees.

Under each prescription group, data were randomly split into a training set (80%), a validation set (10%), and a test set (10%). To avoid contamination of the validation/test sets with training data, visits from the same individual were included only into a single set. The predicted SBP reduction under each prescription type was compared with the true outcome under standard of care and the counterfactual outcome corresponding to following the previous regimen.

## Results

Table 1 summarizes the basic statistics of the entire dataset and the specific subgroups identified. Most of the patients in our dataset are older adults with a mean age of 60.93. The average SBP among all patient visits was above 130 mmHg. The dataset included patients with 10 different races, with the majority being Black patients (49.68%) or White patients (26.10%). The percentage of Black patients is significantly higher than other races. Through all patient visits, ACEi was the most common active prescription. While almost 1/3 of patient visits were on ACEi, only 2.21% of them were on MRA.

**Table 1:** Dataset summary statistics

Features	All patients N = 42,792
Mean age*	60.93
Mean SBP (mmHg)*	136.11
Female	22,445 (52.45% of N)
Male	20,345 (47.54% of N)
White Patients	11,170 (26.10% of N)
Black Patients	21,260 (49.68% of N)
Hispanic Patients	3,946 (9.22% of N)
Ever Cigarette User	11,790 (27.55% of N)
Hypertension drugs used†	All Patient Visits (M = 432,096)
Calcium Channel Blockers (CCBs)	115,450 (26.72% of M)
Thiazide diuretics	92,290 (21.36% of M)
Angiotensin II Receptor Blockers (ARBs)	43,072 (9.97% of M)
ACE Inhibitors (ACEi)	129,800 (30.04% of M)
Beta-Blockers	86,171 (19.94% of M)
Loop diuretics	35,316 (8.17% of M)
Mineralocorticoid Receptor Antagonists (MRAs)	9,550 (2.21% of M)

*Data is calculated through all patient visits

^†^Accounted for both monotherapies and drug combinations

The performance of the prescriptive model is shown in Table 2. Two strategies with different policies to prescribe actions were compared (deterministic vs. randomized). The performance was tested under five different (random) splits of the data into training and test sets. We calculated the mean (over the five splits) SBP reduction under the model recommended prescription and the true reduction under standard of care. All models under two specific strategies outperform the current prescription and standard of care. The best outcome is achieved by DRLR-informed KNN, which can achieve mean SBP reduction of 14.22 mmHg$\pm 0.5$. This amount of reduction is 70.30% better than the SBP reduction under standard of care. The other models also gained better control than the standard prescription with 59.04–22.99% higher SBP reduction compared to the standard of care. However, even the second-best model, OLS-informed KNN, achieved 6.61% lower reduction compared to the best model. This shows the superiority of our robustified DRLR procedure against outliers in terms of the improvement in outcomes. The performance of the randomized strategy did not differ much from the performance of the deterministic one. Still, considering that the randomized rule gives more flexibility for the prescriber to explore suboptimal options in the model and to avoid potential medical contradictions, the randomized policy is preferable.

**Table 2:** The reduction in future Systolic Blood Pressure (SBP) in mmHg

Algorithm	SBP reduction (mmHg)	
	Deterministic	Randomized
LASSO	− 10.71 (0.34)	− 10.73 (0.35)
CART	− 10.24 (0.55)	− 10.28 (0.54)
OLS + KNN	− 13.28 (0.34)	− 13.27 (0.34)
DRLR + KNN	− 14.22 (0.5)	− 14.21 (0.49)
Current regimen	− 8.31 (0.08)	
Standard of care	− 8.35 (0.07)	

Current regimen refers to the predicted outcome if the patient continues on the current prescription in the record. Standard of care refers to true outcome under the regimen prescribed by clinicians; mean (standard deviation)

**Fig. 1:**
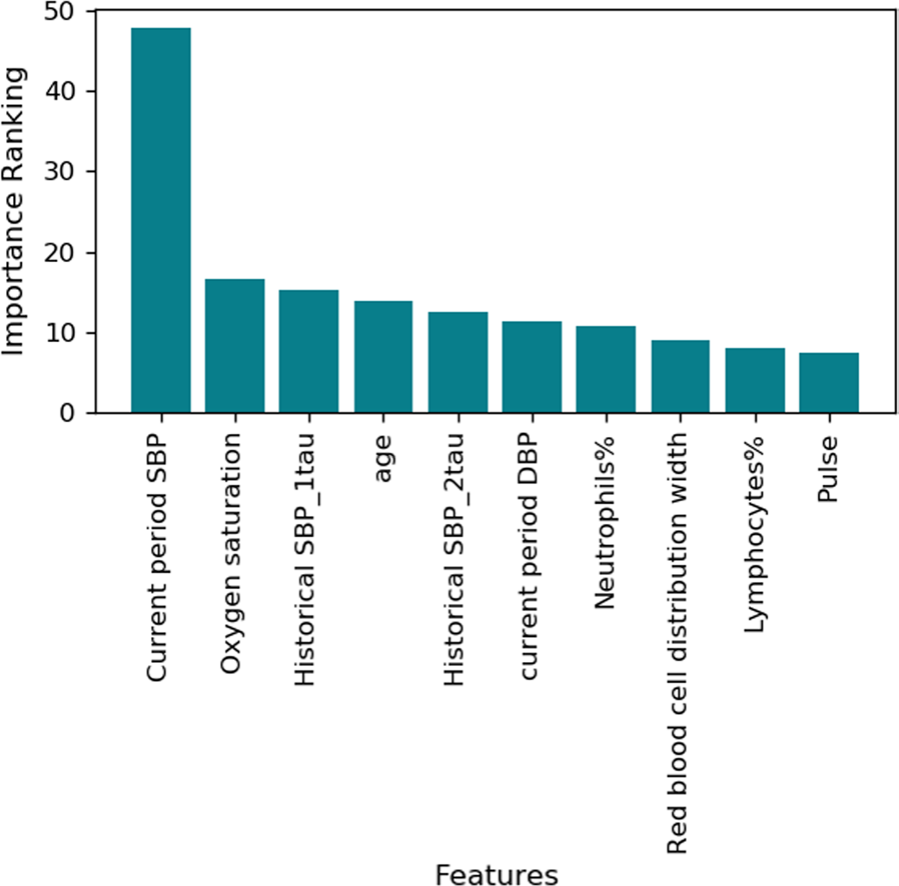
Top 10 predictive features summarized over all the sub-models in DRLR-informed KNN. Historical SBP_1tau and Historical SBP_2tau refer to the Systolic Blood Pressure (SBP) values in previous periods. DBP: Diastolic Blood Pressure. Neutrophils%: Neutrophils as percent of blood leukocytes. Lymphocytes%: Lymphocytes as percent of blood leukocytes

Sub-models were developed for each prescription in the patients’ menu of treatment options. The potential outcome under a particular treatment was estimated by the corresponding sub-model, which is trained on patient visits that were previously on this treatment. We summarized the top 10 predictive features by assembling the feature importance scores (regression coefficient magnitudes) from all the sub-models in our best prescriptive model (DRLR-informed KNN). The results are shown in Fig. 1, with the features shown on the x-axis and the corresponding importance scores shown on the y-axis. All the features in the figure were positively correlated with the future predicted SBP. The most predictive feature is the SBP value in the current period, which has significantly higher importance score than others. The SBP values of the past two periods and the current period DBP also showed great influence on the prediction. Among all the clinical features, ‘Oxygen saturation’ is the most important one in preicting SBP. The larger variation of ‘Oxygen saturation’ may represent those medically fragile patients who have other comorbidities or even acute or subacute illnesses that impact their oxygen saturation. Moreover, the older patients were generally more likely to have high SBP since ‘Age’ is the fourth significant feature with positive correlation with the outcome. There is also evidence supporting the possible roles of neutrophils and Red Blood Cell Distribution Width (RDW) in arterial hypertension and other cardiovascular diseases [35–37]. One explanation is the possible mechanism via the overall inflammatory milieu causing arterial stiffness or subtle changes of myocardial or endocrine function [35].

Since the model is prescribing monotherapy, only 30.68% of the recommendations from the DRLR + KNN algorithm indicated continuing the current prescription, and 28.91% of the algorithmic recommendations matched the standard of care. In the cases where the prescriptive model recommended switching from the given therapy, the average reduction of SBP under algorithmic therapy is 14.65 mmHg, which is 13.48% better than the average reduction achieved by continuing the current medication in the record.

We randomly generated 350 samples from our best prescriptive algorithm (DRLR-informed KNN model), each with the corresponding patient features and the model’s recommended treatment. Examples of generated patients' samples have been provided in Additional file 1. For each recommended prescription with the best SBP outcome, we summarized the corresponding neighborhood profile the model has learnt and used to generate the optimal decision. For example, in the ‘ACEi’ profile, we included all patients who were recommended to take ACEi in the future and summarized the features over all their corresponding neighborhoods found by the model. In this way, we sought to interpret the decision-making process of the model and generalize the patient characteristics that point to a certain prescription type. All these results were validated through a sanity check by BMC clinicians, to ensure that the recommendations from our model are clinically rational. Among the 350 generated cases, 307 of them (87.71%) have passed the sanity check. A number of 19 out of the remaining 43 recommendations were not endorsed due to the nature of monotherapy setting of the model, which reduced the recommendation to one of the drugs from the drug combination in the record. This process is also known as deprescribing, and clinicians decided not to take this into account since it is still rare in the clinic and the effects may vary on a case-by-case basis. The additional cases that did not pass the sanity check had to do with medications that may be used for other reasons (e.g., beta blockers are also used for arrhythmias) and contra-indications the model did not capture.

## Discussion

### Feasibility of deprescribing

Deprescribing refers to the process of tapering off or stopping medications to prevent adverse drug reactions and achieve better health management [38, 39]. Among all the patient visits, 9,196 of them were originally on multiple antihypertensive medication types, where they were able to achieve on average an 8.07 mmHg SBP reduction by following the standard of care. However, following the prescriptive model recommendations, led to a 14.33 mmHg SBP reduction, on average, by monotherapy, which is 77.57% better than the standard outcomes. Among these 9,196 patients on combo drugs, 7,241 of them (78.74%) indeed achieved better SBP reduction than the standard of care. We summarized the profile for these 7,241 patients in Table 3. The mean age of them is 65.05, which is higher than the mean age over the entire dataset (60.93). Compared to other patients, they have a higher chance to have medical contradictions, since more of them have diagnoses of other diseases, including diabetes and chronic systolic heart failure. The percentage of these patients with a diabetes diagnosis is 20.99%, which is higher than the percentage in the entire dataset.

**Table 3:** Summarized statistics of patients with effective deprescribing

	All patient visits	Visits with effective deprescribing
Mean age	60.93	65.05
Diabetes	44.69%	54.07%
Chronic systolic heart failure	2.32%	3.25%
Atherosclerotic heart disease	13.23%	18.86%
Peripheral vascular disease	7.29%	11.02%

Percentages in all patient visits are calculated as a fraction of the total number of patient visits in the test set (N = 31,758). The population in the effective deprescribing cohort consists of patients with better SBP control under deprescribing (M = 7241). All diseases refer to the corresponding diagnosis in ICD9/ICD10 (International Classification of Diseases) codes

The potential benefits shown on deprescribing are interesting, since it is recognized by both the 8th report of the Joint National Committee for the Prevention, Detection, Evaluation and Treatment of Hypertension (JNC8) [40] and the 2013 guidelines of the European Society of Hypertension and the European Society of Cardiology (ESHESC) [41] that patients with SBP 20 mmHg above treatment goals should take combinations of antihypertensive agents for efficient hypertension control. However, deprescribing is increasingly encouraged among patients under control and older people with multi-morbidities, for whom the risk and burden of the drugs may outweigh their effectiveness [39, 42]. In fact, a clinical trial has shown that antihypertensive medication reduction was noninferior compared with the usual care among older patient, with respect to the proportion of patients with SBP lower than 150 mmHg at 12 weeks [43]. Moreover, studies have shown that deprescribing of sedatives and nonsteroidal anti-inflammatory drugs is cost-effective [44, 45]. In fact, the potential harms and long-term benefits of many cardiovascular drugs are still unknown [39], thus, clinicians should tailor appropriate treatment type more carefully, especially with the addition of new medications.

### Neighborhood profile analysis

**Fig. 2:**
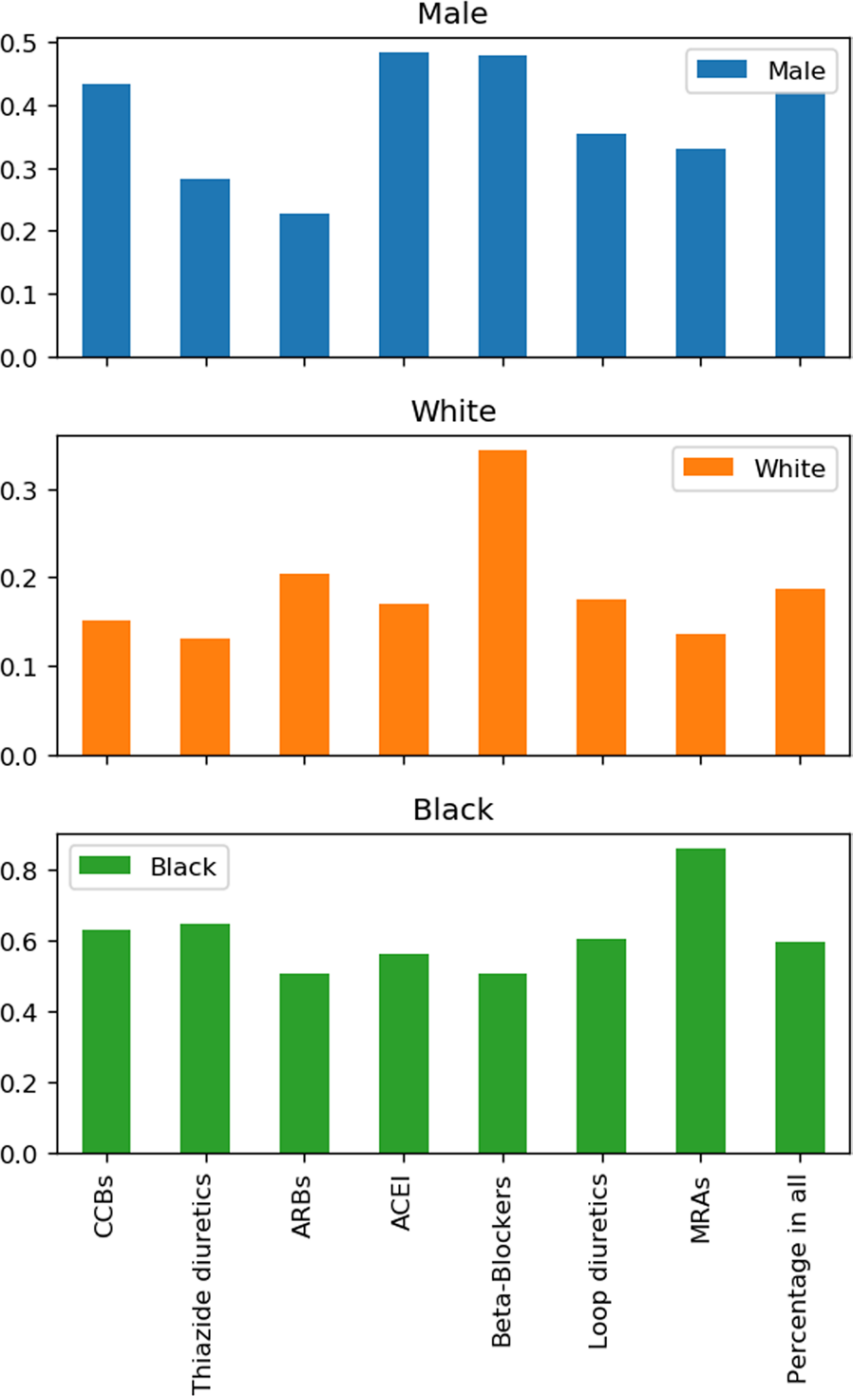
Subgroup analysis in the neighborhood profiles for each recommended prescription type. The y-axis shows the corresponding percentage of certain subgroups in each drug type neighborhood profile. If the percentage of group A in the drug X neighborhood is higher than the overall percentage of A in the whole test set, it implies that group A may generally get better response under drug X. CCBs: Calcium Channel Blockers; ARBs: Angiotensin Receptor Blockers; ACEi: ACE inhibitors; MRAs: Mineralocorticoid-Receptor-Antagonists

Seven neighborhood profiles were built to summarize patients’ characteristics the model has captured for each prescription group. Figure 2 shows the corresponding percentage of certain subgroups in each neighborhood profile. If the percentage of group A in the drug X neighborhood is higher than the overall percentage of A in the whole test set, it implies that group A may generally get better response under drug X. Most of the male patients fit into ACEi and Beta-Blockers, as the corresponding neighborhoods both comprise around 48% of male patients, which is 14.29% higher than the overall male percentage. On the other hand, female patients are more likely to be recommended an ARB since 77.26% of the ARB neighborhood consists of females.

Black patients generally achieved better response on MRAs compared with other medication types, where 86.14% of the MRAs neighborhood consists of Black patients. Whereas White patients do better on a Beta-Blocker, and the percentage of them in the corresponding neighborhood is 83.2%, higher than their overall percentage. Some hypotheses have been put forward to explain the favor of different prescriptions for Black and White patients [46–49]. Nevertheless, race is a social construct that usually comes with other environmental and socioeconomic factors that could be a proxy of unmeasured variables, as opposed to some essential physiologic difference. Thus, it is hard to obtain a clear explanation towards such racial difference.

### Hypertensive treatment initiation for non-diabetic drug naïve patients

**Fig. 3:**
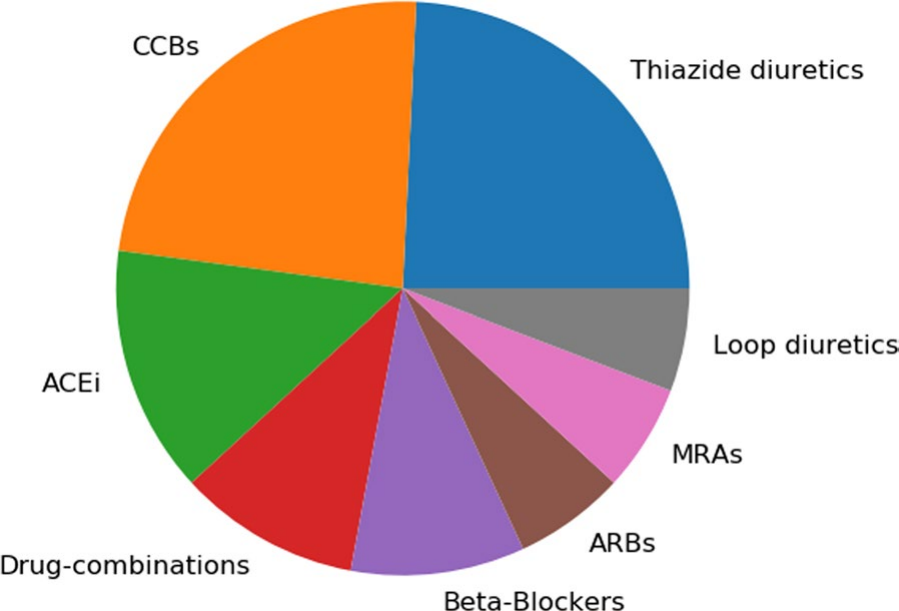
Model recommended prescription type for non-diabetic medication-naïve patients. CCBs: Calcium Channel Blockers; ARBs: Angiotensin Receptor Blockers; ACEi: ACE inhibitors; MRAs: Mineralocorticoid-Receptor-Antagonists

Designed for personalized hypertension treatment, our model aims to make suggestions in real-world scenarios that have true clinical equipoise. One of the cases is the prescribing scenario with non-diabetic and antihypertensive drug naïve patients. We found 7,945 non-diabetic patient visits among the test set who did not take any antihypertensive medication previously. The benefit of using the algorithm amounted to a 15.56 mmHg SBP reduction on average, which is 9.42% higher than the average gain from the model. We summarized the optimal prescription types recommended by the algorithm for these patients in Fig. 3. Among all the therapies, most of the patients were recommended to initialize with monotherapy of Thiazide diuretics or CCBs. This is consistent with the ACC/AHA hypertension treatment guideline, where prescription for drug-naïve patients is recommended to come from one of the four classes: Thiazide diuretics, CCBs, ACEi or ARBs [4]. It has also been proved in randomized trials that CCBs and Thiazide diuretics are more effective as the initial treatment for Black subjects [50, 51]. Interestingly, 9.47% of patients were recommended to take combination therapies, for whom the current averaged SBP (152.50 mmHg) is 12.04% higher than the mean SBP of the whole dataset. The very high blood pressure may explain their requirement of more intense treatment.

### Limitations

Although the model can significantly improve hypertension control on standard of care, this study has several limitations. First, since it is a retrospective study, only the outcomes with administered prescriptions were observed. The counterfactual outcomes under other medications are unknown and can only be estimated by the predictive model. All the results and improvements we showed here are still worth to be tested in a real clinical trial. Second, there are actually a lot of factors aside from the magnitude of SBP reduction that need to be considered in the routine HTN management, including tolerance of past regimens, allergies, dosage for current medications, best medication for other chronic conditions, etc. Given the nature of the dataset, we cannot account for some of these, which could limit pragmatic clinical utility in an all-hypertensive patients use case.

## Conclusions

This study developed a prescriptive model that determines the optimal therapies for patients based on their specific characteristics. Our proposed robustified algorithm DRLR-informed KNN is developed to accommodate outliers in the EHR data, and it achieved 70.30% larger (− 14.22 mmHg$\pm 0.5$) SBP reduction than standard of care, with the results being 7.08% better than the 2nd best model. Neighborhood profiles and patients’ recommendations samples were generated to interpret the decision-making process of the model. 87.71% of the randomly sampled cases passed the professional sanity check by BMC clinicians. The results imply that:

1. personalized hypertension treatment by ML methods can provide good support for medical decision-making, leading to improved drug efficacy;
2. the feasibility of deprescribing may be underestimated since it shows considerable benefits computationally;
3. although developed in limited scenarios, prescriptive algorithm can still provide promising insights on situations with clinical equipoise like drug initializations.

## Supplementary Information

**Additional file 1.** 5 examples of prescription recommendations from 350 generated patients' samples.

## Data Availability

The data that support the findings of this study are available from Boston Medical Center (BMC) but restrictions apply to the availability of these data, which were used under license for the current study, and so are not publicly available. Data are however available from the corresponding authors upon reasonable request and with permission of BMC. All authors read and approved the final manuscript.

## Abbreviations

SBP: Systolic blood pressure
ML: Machine learning
EHRs: Electronic health records
DL: Deep learning
BMC: Boston Medical Center
KNN: K-nearest neighbor
CCBs: Calcium channel blockers
ARBs: Angiotensin receptor blockers
ACEi: ACE inhibitors
MRAs: Mineralocorticoid-receptor-antagonists
SDoH: Social determinants of health
RF: Random forest
DRLR: Distributionally robust linear regression
OLS: Ordinary least square regression
RDW: Red blood cell distribution width
